# ASSOCIATION BETWEEN SUBJECTIVE COGNITIVE DECLINE AND STRENGTH TRAINING IN US ADULTS AGED 45+ YEARS

**DOI:** 10.1093/geroni/igac059.2259

**Published:** 2022-12-20

**Authors:** Matthew Schroeder, Molly Waring, Nicole Fowler, Sherry Pagoto

**Affiliations:** Regenstrief Institute, Inc., Indianapolis, Indiana, United States; University of Connecticut, Storrs, Connecticut, United States; Indiana University School of Medicine, Indianapolis, Indiana, United States; University of Connecticut, Storrs, Connecticut, United States

## Abstract

Subjective cognitive decline (SCD) can be an early marker for Alzheimer’s disease and related dementias. Data supports physical activity to delay cognitive impairment and to improve cognitive functioning. We examined strength training engagement by middle-aged and older US adults with and without SCD. We used data from 121, 059 participated aged 45 years or older from the 2019 Behavioral Risk Factor Surveillance System (BRFSS) from 31 states and Washington, D.C. SCD was assessed by asking participants if they had experienced confusion or memory loss during the past 12 months (yes/no). Participants reported how often they engaged in strength training (e.g., using weight machine, free weights) in the past month. We dichotomized strength training engagement as meeting physical activity recommendations (2+ times weekly) or not (< 2 times weekly). An adjusted logistic regression model, controlling for confounding variables, estimated the likelihood of strength training in relation to SCD. Analyses were weighted; results are nationally representative. SCD was reported by 11.0% (SE: 0.2%) of middle-aged and older US adults. Three in 10 (29.1%; SE: 0.7%) of US middle-aged and older adults who reported SCD engaged in strength training 2+ times a week compared to 34.0% (SE: 0.3%) of US adults without SCD (aOR, 0.9; 95% CI: 0.9-1.0). While middle-aged and older US adults with SCD were less likely to strength train than those without SCD, only a third engaged in recommended strength training regardless of SCD status. Primary care providers should encourage strength training among middle-aged and older adults regardless of cognitive status.

